# Incidence of delirium in three critical care units of a teaching hospital in Brazil

**DOI:** 10.1186/cc10197

**Published:** 2011-06-22

**Authors:** APN Okada, RP Azevedo, FR Machado

**Affiliations:** 1 Universidade Federal de São Paulo, São Paulo - SP, Brazil

## Introduction

Delirium is a disturbance of consciousness in which there is a sharply global deficit of attention associated with change in cognition that cannot be attributed to a pre-existing dementia. Its relevance is not only due to the high incidence, but above all its consequences, such as influence on mortality, morbidity, and prolonging the period of hospitalization.

## Objective

The aim of this study was to assess the incidence of delirium in patients admitted to three intensive care units (ICUs) of a teaching hospital through the diagnostic tool CAM-ICU.

## Methods

The patients were evaluated through the daily application of the CAM-ICU by the same physician. We evaluated the correlation of clinical suspicion of attending physicians, medical residents and nurses to perform the diagnosis of delirium compared with the CAM-ICU, the median time to development of delirium, and risk factors for developing delirium, and compared the outcome between patients who progressed to delirium and those who had no delirium during the study period. Standard descriptive statistics were used. Continuous variables were reported as the mean and standard deviation. Interobserver agreement was assessed using Cohen's kappa statistic (κ). All statistics and their 95% confidence intervals were computed using SPSS software and Medcalc software.

## Results

In the period of 39 days, 106 patients were screened, and 42 patients fulfilled inclusion criteria and were enrolled in the study. The incidence of delirium was 21.4% (nine patients). The average time to development of delirium was 62.67 hours (± 33.76), and 88.9% of patients developed delirium in the first 5 days in the ICU. The agreement of clinical diagnoses in relation to the CAM-ICU method was moderate, with the best agreement assigned to nurses. A trend for increased length of ICU and hospital stay was found between patients who developed delirium. The average time in the ICU for patients with delirium was 12.11 days (± 15.44) and patients without delirium was 5.75 days (± 7.13), *P *= 0.0821. The average time of hospitalization for patients with delirium was 29 days (± 28.99) and without delirium was 21.69 days (± 22.83), *P *= 0.428. See Table 1.

**Table 1:** Correlation of clinical suspicion of attending physicians, medical residents and nurses to perform the diagnosis of delirium compared with the CAM-ICU

κ	Delirium	Hypoactive	Hypoactive or mixed
Attending physicians	0.610	NA	0.009
Medical residents	0.656	0.025	0.035
Nurses	0.690	0.038	0.057

Kappa values for delirium (three subtypes), and only the hypoactive or hypoactive and mixed. NA, not available: no agreement between CAM-ICU and the evaluation by attending physicians.

## Conclusion

Delirium is a common disorder in ICUs. Specific tests should be used regularly in order to optimize the correct diagnosis and treatment of this disturbance.

